# Antigen capsid-display on human adenovirus 35 via pIX fusion is a potent vaccine platform

**DOI:** 10.1371/journal.pone.0174728

**Published:** 2017-03-31

**Authors:** Nadine C. Salisch, Marija Vujadinovic, Esmeralda van der Helm, Dirk Spek, Lars Vorthoren, Jan Serroyen, Harmjan Kuipers, Hanneke Schuitemaker, Roland Zahn, Jerome Custers, Jort Vellinga

**Affiliations:** Janssen Vaccines & Prevention, Leiden, The Netherlands; Swedish Neuroscience Institute, UNITED STATES

## Abstract

Durable protection against complex pathogens is likely to require immunity that comprises both humoral and cellular responses. While heterologous prime-boost regimens based on recombinant, replication-incompetent Adenoviral vectors (AdV) and adjuvanted protein have been able to induce high levels of concomitant humoral and cellular responses, complex manufacturing and handling in the field may limit their success. To combine the benefits of genetic and protein-based vaccination within one vaccine construct and to facilitate their use, we generated Human Adenovirus 35 (HAdV35) vectors genetically encoding a model antigen based on the *Plasmodium falciparum* (*P*. *falciparum*) circumsporozoite (CS) protein and displaying a truncated version of the same antigen (CS_short_) via protein IX on the capsid, with or without a flexible glycine-linker and/or a 45Å-spacer. The four tested pIX-antigen display variants were efficiently incorporated and presented on the HAdV35 capsid irrespective of whether a transgene was encoded or not. Transgene-expression and producibility of the display-/expression vectors were not impeded by the pIX-display. In mice, the pIX-modified vectors induced strong humoral antigen-specific immunity that increased with the inclusion of the linker-/spacer molecules, exceeded the responses induced by the genetic, transgene-expressing HAdV35 vector, and surpassed recombinant protein in potency. In addition, the pIX- display/expression vectors elicited high antigen-specific cellular immune responses that matched those of the genetic HAdV35 vector expressing CS. pIX-modified display-/expression HAdV vectors may therefore be a valuable technology for the development of vaccines against complex pathogens, especially in resource-limited settings.

## Introduction

There is growing interest in recombinant, replication-incompetent Adenoviral (AdV) vectors for genetic vaccination due to their ability to induce strong cellular immune responses against the encoded transgenes [1–3]. While vectors based on human Adenovirus 5 (HAdV5) are most frequently used, their immunogenicity is impaired by high levels of pre-existing immunity [4, 5]. Alternative Adenovirus serotypes such as HAdV35, HAdV26 and ChAdV3 are highly immunogenic while less seroprevalent and unaffected by pre-existing immunity to HAdV5, making them suitable as vaccine vectors [6–8]. AdV vectors characteristically induce strong cellular responses against their encoded transgenes [9–11], compared to recombinant, adjuvanted protein that induces strong antibody responses.[12] Those antibody responses alone may, however not suffice to provide protection against complex pathogens where a synergistic T cell response has proven to be beneficial [13–15].

Heterologous prime-boost regimens are one means by which concomitant cellular and humoral immune responses can be induced. In preclinical studies, priming with HAdV35 or -26 vectors and boosting with adjuvanted, recombinant protein, or vice versa [10, 16], induced increased levels of both humoral and cellular antigen-specific immune responses compared to the individual vaccine components alone.[16] Despite this advantage, complex clinical handling may limit the success of heterologous prime-boost regimens in the field. Display of disease-relevant epitopes on the AdV surface by modifying the capsid proteins could serve as a platform to combine the benefits of AdV-based genetic- and protein-based vaccination within one vaccine construct [17]. While all three major capsid proteins—hexon, penton, and fiber—allow insertion of small heterologous peptides into their highly variable protein regions [18], the 240 copies of the minor capsid protein IX (pIX) [19, 20] tolerate fusion of relatively large, functional proteins to its surface-exposed C-terminus without drastically decreasing its function [21–23]. This so called ‘pIX-display technology’ has been used to influence the cell tropism of the vector [23, 24] or to display antigen on the vector surface [18, 25, 26]. The feasibility of pIX-antigen display on genetic HAdV5 vectors expressing a vaccine transgene has been successfully demonstrated in preclinical vaccination studies for different antigens [25, 26], indicating that AdV-based pIX-display-/expression vectors may be a potent approach to induce humoral and cellular immune responses with a single vaccine vector.

Based on previous observations with a HAdV35 vector that genetically encodes the *Plasmodium falciparum* circumsporozoite (CS) transgene in E1 [27] we generated display-/expression vectors presenting the central, B-cell epitope-rich four-amino acid (NANP) repeat region of the CS protein (CS_short_) as a model antigen on the capsid via pIX. Optimal antigen display was explored by fusing the protein directly to pIX, or via a flexible glycine-linker and/or a 45Å-spacer [24]. We show that all pIX-display variants were efficiently incorporated and presented on HAdV35 vectors with and without the genetically encoded transgene and that transgene-expression or vector yields in the E1-complementing producer cell line remained unaffected. In the mouse model, the unadjuvanted pIX-display vectors induced strong humoral antigen-specific immune responses that were increased by the presence of the linker-/spacer molecules and exceeded those induced by the parental genetic HAdV35 vector only expressing the transgene. Vector potency was higher than that of a recombinant protein control. In addition, the pIX-display/expression vectors elicited high antigen-specific cellular immune responses that matched those of the genetic HAdV35 vector expressing the transgene and that were not achieved by mixing protein with AdV vectors.

## Materials and methods

### Ethics statement

Animal work was performed according to the Dutch Animal Experimentation Act and Guidelines on the Protection of Experimental Animals by the Council of the European Committee (EU Dir. 86/609) after approval by the Dier Experimenten Commissie of Janssen Vaccines under permit numbers CRH0211 and CRH0233.

### Vector design, generation and purification

Recombinant, replication-incompetent, genetically modified Advac HAdV35 pIX-display-vectors with a mammalian-codon optimized *P*. *falciparum* CS protein gene in E1, a Luciferase reporter gene (Luc), or without a transgene in E1, were generated. The CS protein gene is a *P*. *falciparum* 3D7 protein based on the EMBL CAH04007 sequence of which the last 14 amino acids of the C-terminus are truncated (minus the GPI anchor) [27]. The CS_short_ antigen is a fragment of the CS protein consisting of 27 consecutive NANP repeats. Different variants of the pIX-display-vectors, with and without a transgene, were generated by fusing CS_short_ directly to pIX (HAdV35.CS.pIX-CS_short_, HAdV35.empty.pIX-CS_short_), via a 3-amino acid glycine-linker (HAdV35.CS.pIX-Gly-CS_short_, HAdV35.empty.pIX-Gly-CS_short_), via a 45Å-spacer [24] (HAdV35.CS.pIX-45-CS_short_, HAdV35.empty.pIX-45-CS_short_) or a combination of glycine-linker and 45Å-spacer (HAdV35.CS.pIX-Gly45-CS_short_, HAdV35.empty.pIX-Gly45-CS_short_). HAdV35-based vectors with the full-length CS sequence fused to pIX were also generated (HAdV35CS.pIX-CS, HAdV35.empty.pIX-CS). The control vectors included the pIX-unmodified vectors with (HAdV35.CS, and HAdV35.Luc) or without a transgene (HAdV35.empty, HAdV26.empty and HAdV5.empty), a HAdV35-based luciferase-expressing vector without the pIX protein (HAdV35.Luc.ΔpIX), and a luciferase-vector carrying a pIX-modification (HAdV35.Luc.pIX-Gly-CS_short_).

HAdV35 Advac vectors were generated by homologous recombination of three plasmids in which the right end of the genome ((pIX-deleted pWE.Ad35.ΔpIX.EcoRV, digested with NotI and EcoRV), pBr.Ad35 PRdE3 5E4orf6/7 (digested with PacI and NotI) and the left end of the genome,[28] (pAdapt35.Bsu.pIX-mod, digested with PacI) were transfected into PER.C6 cells. The vectors (S1 Table) were plaque-purified and up-scaled on PER.C6 cells at 37°C/10% CO_2_ in Dulbecco’s modified Eagle’s medium supplemented with 10% fetal bovine serum (FBS; Life Technologies Inc.) and 10 mM MgCl_2_. Vectors were purified by two-step CsCl-gradient centrifugation, dialyzed in formulation buffer and tested for viral titers. The physical viral particle concentration (VP/ml) was determined by optical density (OD) in the presence of SDS [29] and the infectious units (IU/ml) were titrated by TCID_50_ (tissue culture infectious dose 50%), followed by calculation of the viral particles to infectious units (VP/IU) ratios and the productivity expressed in viral particles per cm^2^ of production flasks (VP/cm^2^).The purified vector batches were tested for Bioburden and Endotoxin levels (Milipore).

### Capsid-incorporation of modified pIX-CS_short_ and transgene-expression

Surface-display of different pIX-modifications was determined by Western Blot of purified HAdV35 particles. For CS transgene-expression, A549 cells were transduced with 5000 VP/cell and incubated for 48 hours at 37°C/10% CO_2._ The cells were harvested and lysed using a lysis buffer (Janssen) supplemented with complete protease inhibitor (Roche) and Benzonase (EMD Milipore). For pIX capsid-incorporation and CS transgene-expression, the pIX-display-vectors were compared to the control vectors HAdV35.empty and HAdV35.CS. Purified viral particles (VP/well) or A549 cell lysates were denatured and reduced in protein loading buffer with reducing agent (Invitrogen) at 70°C, then separated on pre-cast 4–12% Bis/Tris Nu-PAGE gel (Invitrogen) in MOPS buffer (Invitrogen) at 175 Volt, 500 milli-Ampere. The protein was transferred to a nitrocellulose membrane according to manufacturer’s recommendations using iBlot Transfer stacks (iBlot system; Invitrogen). Protein staining was performed for 1 hour with antibodies specific for CS (2A10) or pIX (6740, monoclonal antibody generated in-house), or loading control antibodies specific for the Adenovirus fiber protein (AdV5 4D2, Abcam) for viral particles, or βActin (AC-15, Abcam) for cell lysate, in 5% non-fat dry milk (BioRad)/Tris buffered Saline Tween 20 (Invitrogen). The protein was visualized by staining with fluorescently labeled IRDye800CW/680RD 1:10000 goat anti-mouse/rabbit and recorded on an Odyssey Infrared Imaging System (Li-Cor).

### pIX-CS_short_ surface-display by Electron Microscopy

Capsid-display of the pIX-modifications was confirmed with Electron Microscopy (EM). Purified pIX modified vectors were coated with the primary anti-CS antibody (clone 2A10) on a copper grid with a carbo-coated Formvar film and incubated for 60 minutes at room temperature (1:1). After washing, the grids were stained with gold labeled anti-mouse Protein A Gold 10 nm (PAG10) 1:200 in 1 mM PBS containing 2% bovine serum albumin (BSA) and 0.1% Tween 20 buffer for 60 minutes at room temperature and fixed using 1.5% glutaraldehyde in cacodylate buffer. Samples were subsequently negatively stained with 2% Silicotungstic acid (STA) and visualized with a transmission electron microscope (FEI Tecnai 12 BioTwin).

### Determination of capsid stability

Capsid stability was determined by a Heat Stability luciferase-based assay [23, 30]. Luciferase-encoding vectors containing a pIX-CS_short_ modification were incubated in parallel with HAdV35.Luc as a positive reference, and HAdV35.Luc.(Δ)pIX at 45°C for up to 20 minutes. At 2-minute intervals, samples were taken and transferred to A549 cell cultures at 500 VP/cell. After 48 hours, cells were cryolysed in lysis buffer with 1mM dithiothreitol prior to the addition of luciferase substrate (Promega). The luciferase activity was measured on a Luminoskan microplate luminometer (Thermo Scientific).

### Production and characterization of the *P*. *falciparum* CS protein

CS protein of the same sequence as in the HAdV-vectors was produced in the methylotropic yeast strain *Hansenula polymorpha* RB11 clone by ARTES Biotechnology GmbH (Germany). A C-terminal His tag sequence was introduced into the construct to facilitate Ni-column purification of the CS protein. The protein was characterized as previously described.[10]

### Mice and immunizations

Six- to eight-week old, specific pathogen-free female BALB/c (H-2D) mice were purchased from Charles River (L’Arbresle-Cedex, France) and kept at the AAALAC-certified institutional animal facility under specified pathogen-free conditions. Animals were co-housed per group in IVC cages using compressed sawdust as bedding, under controlled conditions of temperature, humidity and light (12-hour light, 12-hour dark cycles). Standard rodent diet and water were provided ad libitum and mice were provided with nesting material and enrichment. Animal wellbeing was checked daily and pre-set humane endpoints were used to define study-unrelated sacrifice criteria by a veterinarian. All measures were taken to minimize pain, distress and suffering and all procedures were performed by trained personnel. Mice were vaccinated intramuscularly in the quadriceps of both hind legs (50 μl/leg) with the indicated vector particle- or protein-doses in formulation buffer, under isoflurane anesthesia. Serum was obtained by submandibular bleeding throughout the study or by heart puncture under isoflurane anesthesia at the end time points, during which spleens were aseptically removed after cervical dislocation.

### Determination of CS-specific antibodies by IgG-ELISA

*P*. *falciparum* CS protein-specific total IgG, or subclass-specific IgG1 and IgG2a in serum were determined by an enzyme-linked immunosorbent assay (ELISA) as previously described [27]. Relative serum titers of total IgG (ELISA units/ml) were calculated in comparison to a *P*. *falciparum* CS-specific reference serum using a 4-parameter curve fit model. The IgG1- and IgG2a-specific measurements were used directly to calculate the ratio between IgG2a and IgG1, by dividing the reciprocal dilution at which the OD_492_ reached three times that of the background measurement in a naïve control sample.

### Determination of CS- and HAdV35 hexon-specific cellular immunity by IFNγ-ELISPOT

Antigen-specific cellular immunity in vaccinated mice was assessed using an interferon-gamma (IFNγ) enzyme-linked immune-spot assay (ELISPOT) as previously described [27]. Freshly isolated splenocytes were incubated either with a pool of 15-mer peptides overlapping by 11 amino acids, spanning the entire sequence of the *P*. *falciparum* CS protein, or with the described H-2Kd-MHC class I-restricted, immunodominant HAdV35 hexon epitope KYTPSNVTL. The peptide pool and single peptide were used at a final concentration of 1 ug/ml for each individual peptide. The numbers of spot-forming units (SFU) per 10^6^ cells were calculated.

### Indirect immunofluorescence assay

The binding of vaccination-induced antibodies to the native CS protein on the surface of *P*. *falciparum* sporozoites coated on glass slides (~5000 plasmodia/ well; Radboud University Medical Center, Nijmegen, The Netherlands) was evaluated using an indirect immunofluorescence assay (IFA) as previously described [27]. Sera obtained from animals immunized with HAdV35.empty were used as negative control for IFA specificity, the CS-specific monoclonal antibody clone 2A10 as a positive control.

### Statistical analysis

ELISA Units/ml and SFU/10^6^ cells were log-transformed and group comparisons performed using ANOVA models. For repeated measurements over time, a random intercept was added to the ANOVA model to account for correlated observations. Comparisons between groups containing values below the lower limit of quantification were analyzed using censored regression models. Correction for multiple testing was applied using the Dunnett method, since there was a fixed reference group in each analysis. Differences with a p≤0.05 were considered significant. All statistical analyses were performed using SAS software, version 9.2 (SAS Institute Inc., 2011).

## Results

### Generation of HAdV35 pIX-display-vectors

**Fig 1:**
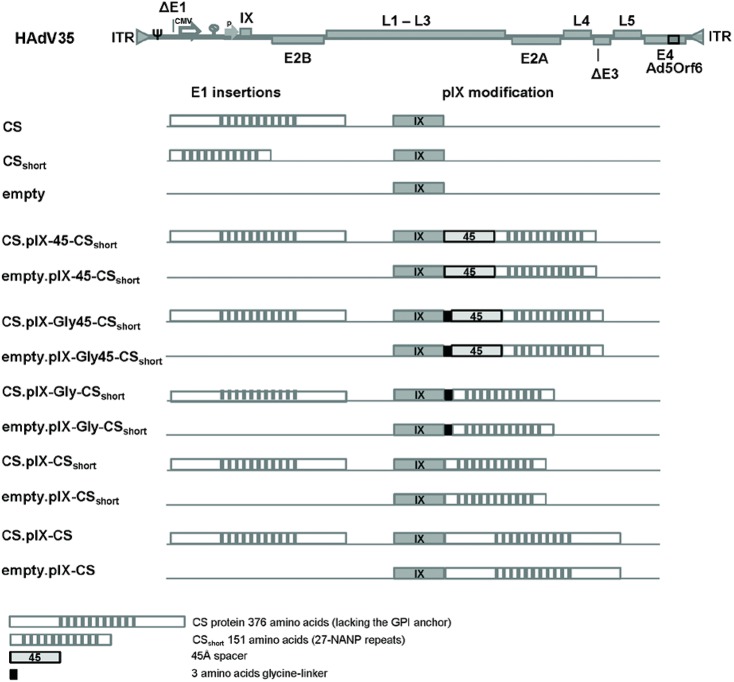
Design of HAdV35 pIX-display-vectors Schematic representation of pIX-display HAdV35 Advac vectors containing a human CMV promoter and SV40 poly-A expression cassette in E1 and the native pIX promoter (P). The vectors either express a 376 amino acid CS protein (lacking the GPI anchor) or no transgene (empty) in the E1 region. The pIX-display-vectors, with and without the CS-transgene in E1, are genetically modified to display the 151 amino acid CSshort with or without a 3 amino acid glycine-linker (Gly) and/or 45Å-spacer.

To evaluate pIX-display technology as a vaccine platform, we generated a panel of recombinant, replication-incompetent HAdV35 vectors, displaying a model antigen consisting of a truncated version of the *P*. *falciparum* CS protein (CS_short_) on pIX. To optimize the display design for B-cell responses, the panel included empty vectors in which CS_short_ was either directly fused to the C-terminus of pIX, or indirectly using an alpha-helical 45Å-spacer,[24] a glycine-linker for additional flexibility, or both combined (“pIX-display-vectors”). All four pIX-CS_short_ constructs were also implemented on HAdV35 vectors encoding the CS protein as a transgene in the E1 region (“pIX-display-/expression vectors”). To assess potential size-limitations of the pIX-fusion, we generated HAdV35 display- and display-/expression variants in which full-length CS protein was fused to pIX. Vector designs are shown in Fig 1.

### All pIX-CS_short_ variants are incorporated into the capsid of pIX-display- and display-/expression vectors

**Fig 2:**
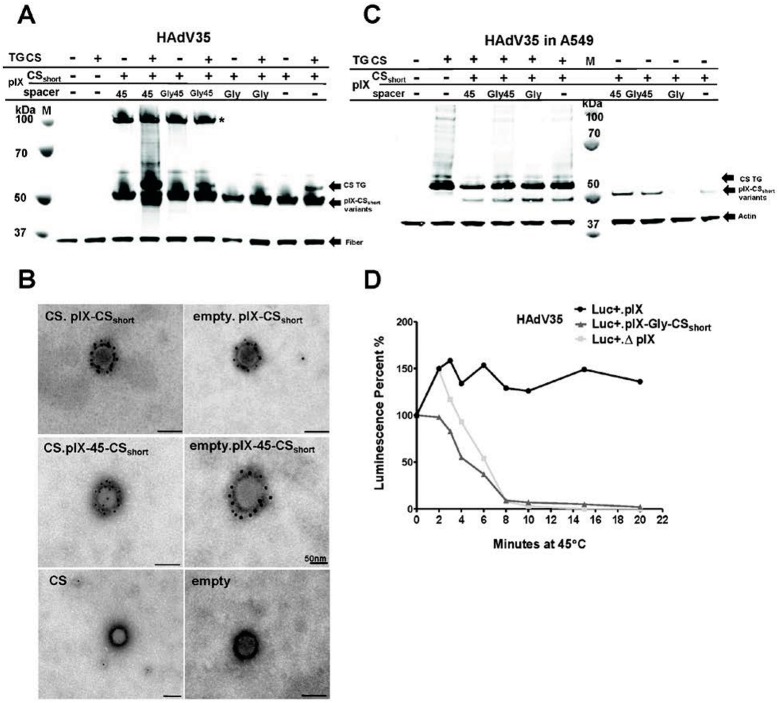
Characterization of the pIX-CS_short_ display- and display-/ expression vectors. (A) pIX-CS_short_ capsid incorporation in purified HAdV35 vector preparations. To confirm capsid incorporation of the pIX-CS_short_ (~50 kDa variants) 5 x10^9^ VP/well of each purified HAdV35 vector preparation was analyzed by Western blot using anti-CS antibody. To ensure equal loading, the blots were stained with anti-fiber 4D2 antibody (~35 kDa). The pIX-fusion proteins migrate higher than their predicted size in kDa due to the NANP-repeat in the CS protein. Marker (M) is indicated with the corresponding kDa band size. An additional band (~100 kDa) is indicated with an asterisk (*). (B) pIX-CS_short_ capsid display by electron microscopy (EM) anti-CS staining and gold-label staining. Representative EM images of HAdV35.empty.pIX-CS_short_, HAdV35.CS.pIX-CS_short_ (upper row), HAdV35.empty.pIX-45-CS_short_, HAdV35.CS.pIX-45-CS_short_ (middle row), HAdV35empty, and HAdV35 (bottom row) vectors. The bars represent 100 nm unless stated otherwise. (C) *In vitro* expression of CS transgene under non-replicating conditions in A549 cells. Western Blot analysis of cell lysates from A549 cells infected with 5000 VP/cell, using an anti-CS antibody. From left to right, the two controls HAdV35.empty and HAdV35.CS followed by the pIX-display vector variants with a CS transgene in E1 and without the transgene. Marker (M) is indicated with the corresponding kDa band size. (D) Heat Stability Assay showing percent (%) variation in luminescence in lysate of A549 cells infected with HAdV35.luc vector preparations subjected to 45°C temperature stress for 0 to 20 minutes. The percent variation was determined relative to the respective baseline time point (0 minutes).

To evaluate capsid-incorporation, Western blot analyses were performed on purified viral particles of all pIX-display and display-/expression vectors. As evident by anti-CS-specific antibody detection, all pIX-CS_short_ display variants were successfully incorporated into the capsid of display- and display-/expression vectors and detected at similar levels (~50 kDa Fig 2A). In contrast, the pIX-CS fusion variant was not incorporated into the capsid of the HAdV35 display- or display-/expression vectors (data not shown) and excluded from further analysis. The genetically encoded CS protein was detected as an additional band at ~55kDa in purified viral particle preparations, suggesting co-purification or association of transgene product expressed during vector production in the producer cells. The highest level of this additional ~55kDa band was detected in the 45Å-spacer-containing vectors, whereas the lowest levels were observed for the pIX-Gly-CS_short_ display-vector (Fig 2A). In the purified vectors containing the 45Å-spacer, a third band migrating at ~100 kDa was present both in the anti-CS as well as anti-pIX-specific staining, indicating the presence of a pIX-45-CS_short_ dimer (Fig 2A). Anti-pIX staining showed that vector particles contained comparable levels of the pIX-45-CS_short_, pIX-Gly45-CS_short_ and non-modified pIX (S1 Fig).

EM analysis with CS-specific staining confirmed capsid-display of the CS_short_ model antigen on all HAdV35 display- and display-/expression vector particles (Fig 2B and S2 Table).

### pIX-CS_short_ modification does not affect in vitro expression of CS protein from the HAdV35 display-/expression vectors

Using Western blot, no differences in the expression level of CS were observed between the pIX-unmodified HAdV35.CS control vector and the four pIX-CS_short_ display-/expression vectors in A549 cells (Fig 2C). Additional bands migrating at a size corresponding to the pIX-CS_short_ variants (~50 kDa) in both the pIX-modified display- and display-/expression vectors indicate some expression of pIX-CS_short_ modifications under non-replicating conditions, similar to previously reported observations [31].

### Fusion of CS_short_ to pIX reduces intrinsic capsid stability but has no effect on viral titers

To evaluate the impact of the pIX-CS_short_ modification on capsid stability of the vector particles, a luciferase-based heat stability assay was used [23]. In contrast to the non-modified control vector, both the vectors lacking pIX (HAdV35.Luc.ΔpIX) and the pIX-CS_short_ vectors showed rapid decline in luciferase expression, indicating capsid instability (Fig 2D, shown for representative vector HAdV35.Luc.pIX-Gly-CS_short_). The physical viral particles (range 2.5x10^11^-2.5x10^12^ VP/ml) and infectious units (range 1.5x10^10^-3.5x10^11^ IU/ml) and the corresponding VP/IU ratios (range 3–19 VP/IU) were comparable for the eight HAdV35 pIX-modified vector preparations and the non-modified pIX control vectors HAdV35.empty and HAdV35.CS (S2 Table). The productivity (VP/cm^2^) was equally unaffected compared to the non-modified pIX control vectors (S2 Table). These observations suggest that despite reduced capsid stability, the fusion of CS_short_ to pIX did not affect vector producibility.

### pIX-CS_short_ display induces strong CS-specific humoral immune responses that are increased by the presence of a spacer

**Fig 3:**
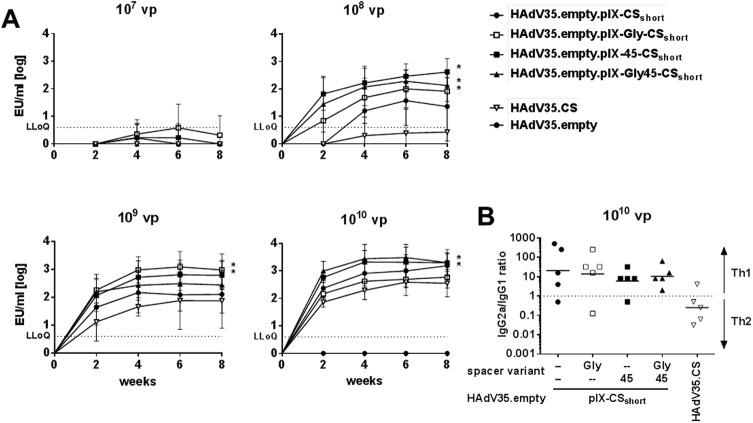
pIX-CS_short_ display induces strong CS-specific humoral immune responses that are increased by the presence of a spacer. (A) Total CS-specific IgG titers in the serum of Balb/C mice over 8 weeks post-immunization with 1x10^7^, 1x10^8^, 1x10^9^, or 1x10^10^ VP/animal of the HAdV35 vectors indicated in the legend. Symbols depict means (n = 5 animals per group and time point), error bars indicate standard deviation, EU indicates ELISA units. Statistical significance was determined using a mixed-model analysis with Dunnett correction for multiple comparisons on log-transformed data per dose across time points. Asterisks indicate statistical significance (p ≤ 0.05). (B) Ratio of CS-specific serum IgG2a to IgG1 levels, 8 weeks after immunization with 1x10^10^ VP of the HAdV35 vectors indicated in the legend. Horizontal bars depict mean ratios.

Immunogenicity of the four HAdV35 pIX-CS_short_ display-vectors was determined in Balb/C mice at a dose range of 1x10^7^ to 1x10^10^ VP/mouse. At 1x10^7^ VP/mouse, none of the tested constructs mounted detectable CS-specific IgG responses (Fig 3A). At 1x10^8^ all four constructs displaying a pIX-CS_short_ construct induced responses by week 4 post-immunization that were maintained until week 8. HAdV35.CS showed comparatively lower potency, with the lowest dose to induce CS-specific IgG responses being 1x10^9^ VP. Titers elicited by the four pIX-CS_short_ modified display-vectors were higher than those induced by the HAdV35.CS expression-vector (significant for titers 1x10^8^ - 1x10^10^ VP. See S3 Table for p-values). CS_short_ display on any of the three linker/spacer constructs further increased immunogenicity, resulting in an earlier onset of responses compared to the direct pIX-CS_short_ fusion construct at 1x10^8^ VP (S3 Table), and titers that were significantly higher than those induced by HAdV35.CS. Although the three different linker/spacer constructs induced CS-specific IgG titers of comparable magnitude, HAdV35.empty.pIX-45-CS_short_ was the only vector to consistently reach responses that significantly exceeded HAdV35.CS at 1x10^8^-1x10^10^ VP (S3 Table).

We analyzed the subtypes of the CS-specific IgGs to understand whether pIX-display of CS_short_ and the usage of the linker/spacer constructs would introduce a Th1 or Th2 bias, with IgG1 indicative of a Th2- and IgG2a of a Th1-type response in this mouse model. Using sera from animals immunized with 1x10^10^ VP of all vectors, we saw that HAdV35.CS immunization induced an overall balanced response, while all pIX-CS_short_ modified vectors induced a stronger IgG2a bias (Th1), independently of whether a linker or spacer was present (Fig 3B).

Taken together, pIX-display of CS_short_ on empty HAdV35 vector particles proved to be highly immunogenic, with the presence of a spacer construct further increasing vector potency.

### CS-specific antibodies induced by pIX-CS_short_ displayed on HAdV35 vectors recognize native CS on *P*. *falciparum* sporozoites

Pooled sera from animals immunized with 1x10^10^ VP of each of the four pIX-CS_short_ modified vectors or of HAdV35.CS as positive reference were equally capable of binding unfixed sporozoites, as determined by IFA with *P*. *falciparum* sporozoite-coated slides (S2 Fig).

### pIX-CS_short_ display on CS-transgene expression- vectors induces strong humoral and cellular immune responses

**Fig 4:**
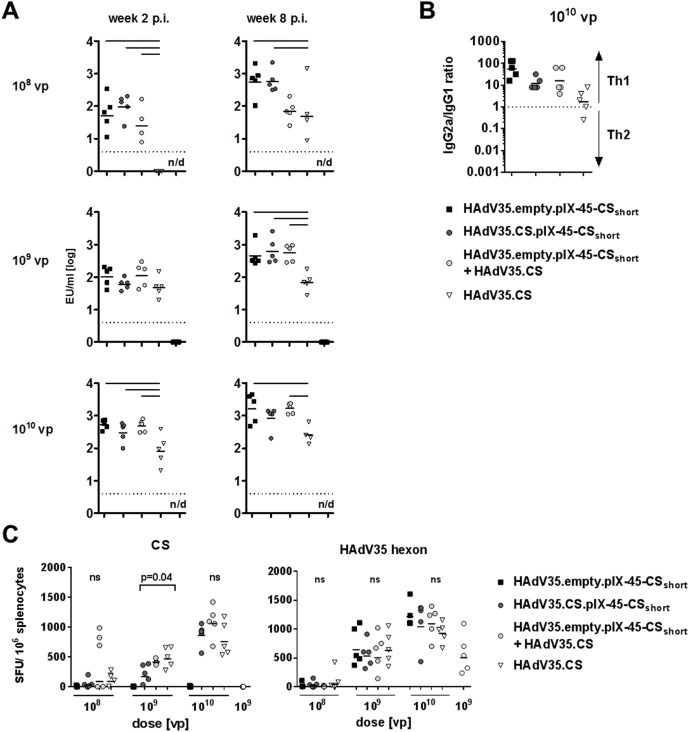
pIX-CS_short_ display on CS-transgene expression-vectors induces strong humoral and cellular responses. (A) Total CS-specific IgG titers in the serum of Balb/C mice 2 and 8 weeks post-immunization with 1x10^8^, 1x10^9^, or 1x10^10^ VP/animal of HAdV35.empty.pIX-45-CS_short_, HAdV35.CS.pIX-45-CS_short_, a mix of HAdV35.empty.pIX-45-CS_short_ and HAdV35.CS, HAdV35.CS alone, or HAdV35.empty (10^9^ VP/ animal only). n = 5 per group. Statistical significance was determined using a two-way ANOVA test with Dunnett correction for multiple comparisons on log-transformed data by time point and dose. Black horizontal bars indicate statistical significance (p ≤ 0.05). (B) Ratio of CS-specific serum IgG2a to IgG1 levels, 4 weeks after immunization with 1x10^10^ VP of the HAdV35 vectors indicated in the legend. Horizontal bars depict mean ratios (n = 5 animals/ group). (C) IFNγ ELISPOT responses to stimulation of splenocytes of Balb/C mice 8 weeks post-immunization with *P*. *falciparum* CS peptide pool (left panel; “CS”) or the H-2Kd restricted immunodominant HAdV35 hexon epitope KYTPSNVTL (right panel; “HAdV35 hexon”). n = 5 animals/ group. Statistical significance was determined using a two-way ANOVA test with Dunnett correction for multiple comparisons on log-transformed data. ns = not significant.

We assessed whether implementation of pIX-display of the B-cell epitope-carrying CS_short_ antigen on HAdV35 encoding the CS protein sequence (including the H2kD-restricted CD8+ T-cell epitope NYDNAGTNL) would maintain the induction of strong humoral immune responses, concomitantly with strong T-cell responses against the encoded transgene. Animals were immunized with 1x10^8^ to 1x10^10^ VP/mouse of HAdV35.CS that either did or did not carry the pIX-45-CS_short_ construct (HAdV35.CS.pIX-45-CS_short_ and HAdV35.CS), or with HAdV35.empty.pIX-45-CS_short_ vector as a reference for the humoral immune response. To assess whether any potential interference between pIX-fusion technology and transgene-expression would be immune-mediated, animals were immunized with a mix of HAdV35.empty.pIX-45-CS_short_ and HAdV35.CS (1x10^8^ to 1x10^10^ VP/ mouse of each of the two vectors; final vector dose of 2x10^8^ – 2x10^10^ VP/mouse). In all remaining groups, the total VP number was adjusted to the total vector dose of 2x10^8^ – 2x10^10^ VP/mouse using HAdV35.empty. In contrast to the expression-vector HAdV35.CS, the display-vector HAdV35.empty pIX-45-CS_short_, and the display-/expression vector HAdV35.CS.pIX-45-CS_short_ induced measurable CS-specific antibody responses at the lowest dose (1x10^8^) 2 weeks post-immunization, indicating higher potency of the vectors compared to the pIX-unmodified expression-vector HAdV35.CS (Fig 4A).

In addition, the titers mounted by 1x10^10^ VP of HAdV35.CS.pIX-45-CS_short_ and HAdV35.empty pIX-45-CS_short_ at week 2 markedly exceeded those induced by HAdV35.CS (p ≤0.005 for all comparisons, Fig 4A). Although all titers increased in magnitude over time, those induced by the expression-vector HAdV35.CS remained significantly lower than those induced by the two pIX-45-CS_short_ fusion constructs at week 8 post-immunization (p≤0.005 for all comparisons in the groups receiving 1x10^8^ and 1x10^9^ VP, Fig 4A). The mix of the display-vector HAdV35.empty.pIX-45-CS_short_ with the expression-vector HAdV35.CS also resulted in significantly increased CS-specific titers at all doses and time points, except at the lowest dose, 1x10^8^. This indicates that immune responses against the transgene do not interfere with the induction of humoral responses against the pIX-CS_short_ fusion construct when provided to the immune system on separate vectors. Calculation of the IgG2a/IgG1 ratio showed that the Th1 bias induced by pIX-CS_short_ on HAdV35.empty vectors was maintained on the HAdV35.CS pIX-45-CS_short_ display-/expression vector (Fig 4B).

Next, CS-specific cellular immune responses against all four constructs were assessed by IFNγ ELISPOT at week 8 post-priming (Fig 4C). As expected, due to the absence of the immunodominant T-cell epitope NYDNAGTNL in the CS_short_ sequence, HAdV35.empty pIX-45-CS_short_ did not induce a response. All other immunization regimens induced responses that followed a dose-response relationship. IFNγ levels induced by the display-/expression vector HAdV35.CS.pIX-45-CS_short_ did not significantly differ from those induced by the expression-vector HAdV35.CS at 1x10^8^ and 1x10^10^ VP, but were slightly reduced at the intermediate dose of 1x10^9^ VP (p = 0.04). IFNγ levels induced by the mix of HAdV35.empty.pIX-45-CS_short_ and HAdV35.CS did not differ from those induced by HAdV35.CS at any of the given doses. To exclude that differences in CS-specific immune responses were caused by slight variations in vaccine dosing, we measured IFNγ-producing T-cells targeting the immunodominant H-2Kd restricted epitope KYTPSNVTL in HAdV35 hexon, which, as expected, did not differ between groups immunized with the same dose (Fig 4C).

These results indicate that CS_short_ display on pIX can be implemented on CS-transgene-expressing HAdV35 vectors, significantly increasing humoral immune responses while maintaining strong cellular immune responses.

### Immunogenicity of pIX-CS_short_ display is higher than that of CS protein and comparable levels cannot be achieved by mixing AdV vectors and protein

**Fig 5:**
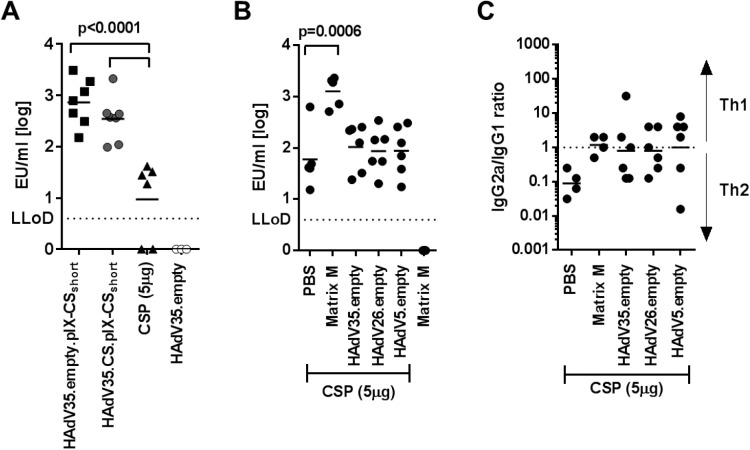
Immunogenicity of pIX-CS_short_ display is higher than that of CS protein and comparable levels cannot be achieved by mixing AdV vectors and protein. (A) Total CS-specific IgG titers in the serum of Balb/C mice 4 weeks post-immunization with 1x10^10^ VP/animal of HAdV35.empty.pIX-CS_short_, HAdV35.CS.pIX-CS_short_, with 5μg of CS protein in PBS, or with 1x10^10^ VP/animal HAdV35.empty. n = 7 per group. Statistical significance was determined using a two-way ANOVA test with Dunnett correction for multiple comparisons on log-transformed data. (B) Total CS-specific IgG titers in the serum of Balb/C mice 4 weeks after immunization with 5μg CS protein in PBS, or mixed with 1x10^10^ VP/animal of empty AdV-vectors (HAdV35, HAdV26, or HAdV5), animals injected with Matrix-M only serve as control. n = 6 per group. Statistical significance was determined using a two-way ANOVA test with Dunnett correction for multiple comparisons on log-transformed data. Ratio of CS-specific serum IgG2a to IgG1 levels, 4 weeks after immunization with 5μg CS protein in PBS only, or mixed with Matrix-M, or 1x10^10^ VP/animal of empty Ad-vectors (HAdV35, HAdV26, or HAdV5). Horizontal bars depict mean ratios (n = 5 animals/ group).

Due to its high potency, recombinant CS protein [10] can be considered a benchmark for the induction of humoral immunity. To compare the immunogenicity of pIX-CS_short_ HAdV35 display-vectors to that of CS protein, we immunized Balb/C mice with 1x10^10^ VP of either the display-vector HAdV35.empty.pIX-CS_short_, the display-/expression vector HAdV35.CS.pIX-CS_short_, or with 5μg of unadjuvanted CS protein (Fig 5A). Animals immunized with 1x10^10^ VP of HAdV35.empty served as a negative control. 5μg CS protein per mouse was chosen since titrations of this CS protein preparation have shown that CS-specific IgG titers plateaued at a dose of 5μg/mouse in an adjuvanted [10] or unadjuvanted setting (data not shown). This dose contains the calculated equivalent of 1.88μg CS_short_ (the CS_short_ sequence consists of the central 27 NANP-repeats and accounts for 37.6% of the CS full-length protein sequence), approximately 30 times more than the theoretical amount of 0.06μg CS_short_ contained in the 1x10^10^ VP of HAdV35.empty.pIX-CS_short_ preparation, assuming full decoration of all 240 pIX- molecules per capsid with the 15.1 kDa CS_short_ modification. At week 4 post-immunization, both pIX-CS_short_-modified vectors induced CS-specific total IgG titers that greatly exceeded those induced by CS protein (p≤0.0001), indicating that presentation of the CS_short_ antigen on viral particles is a potent platform, even compared to the highly immunogenic CS protein.

To determine whether the comparatively high immunogenicity of the pIX-CS_short_ modified vector particles was mediated by an inherent adjuvant-effect of the AdV backbone [32], we immunized Balb/C mice with 5μg CS protein alone or mixed with 1x10^10^ VP of HAdV35.empty and determined CS-specific total IgG levels 4 weeks later (Fig 5B). As a reference point, we chose 5μg CS protein adjuvanted with Matrix-M, and Matrix-M alone as negative control. CS protein adjuvanted with Matrix-M induced CS-specific IgG titers that significantly exceeded those induced by CS protein alone (p = 0.0006) and reached levels similar to those observed in animals immunized with 1x10^10^ VP of pIX-CS_short_ modified HAdV35 vectors (Fig 5A). By contrast, the mixture of CS protein and HAdV35.empty induced only slightly increased titers compared to CS protein alone (p = 0.08). Similarly, ad-mixing 1x10^10^ VP of HAdV26.empty or HAdV5.empty, induced slightly, but not significantly increased IgG titers, excluding a serotype-specific difference in the adjuvant capability of AdV particles in this setting. Mixing of CS protein with Matrix-M or any of the AdV serotypes did, however, produce a more pronounced shift towards a Th1-type response compared to CS protein alone (Fig 5C), which was similar to that observed with pIX-CS_short_ modified vectors (Figs 3B and 5B). Inherent AdV-mediated adjuvation is therefore not likely to be the decisive mediator of the strong immunogenicity of pIX-CS_short_ modified vectors, confirming that display of the antigen on the surface of the vector particles is an essential aspect that mediates the high immunogenicity of the pIX-modified vector platform.

## Discussion

We describe the development and immunological evaluation of replication-incompetent HAdV35 vectors displaying a truncated version of the *P*. *falciparum* CS (CS_short_) as a model antigen via the small capsid protein IX. Multiple pIX-antigen display variants were efficiently incorporated into, and presented on the HAdV35 viral capsid without affecting vector yields. The pIX-display vectors induced strong humoral antigen-specific immunity that increased with the inclusion of linker-/spacer molecules and exceeded the responses induced by a genetic HAdV35 vector expressing a transgene. The potency of the display-/expression vectors surpassed that of recombinant protein control. pIX- display could successfully be implemented on transgene expressing HAdV35, resulting in significantly increased antigen-specific humoral responses comcomitantly with strong cellular immune responses that were not achieved by mixing the protein with Adenoviral vectors.

As reported, pIX can tolerate relatively large fusions of functional proteins (30–120 kDa) to its C-terminus [21, 22, 25, 26]. The finding that the ~41 kDa (376 a.a.) CS protein cannot be incorporated into the capsid was therefore unexpected. This suggests that factors other than size may influence the extent of successful capsid-incorporation, and that empirical selection of protein antigens for pIX antigen fusion might be necessary. Similarly, a vector encoding CS_short_ as transgene failed to express the CS_short_ protein (RNA synthesis confirmed but protein not detected, data not shown), suggesting that in some instances the fusion to pIX could be beneficial in stabilizing the (artificial) antigen.

Some CS antigen-specific observations were made, which may not necessarily be translated to other antigen combinations using the pIX- display/ expression technology. The detection of an additional 55 kDa CS-staining band in all four preparations of HAdV35 vectors encoding CS as a transgene suggest that co-purified CS-transgene expressed during vector production may physically associate with the pIX-display vectors. Proteins containing NANP-repeats demonstrate a high nonspecific association [33], which may also explain the presence of the additional band migrating at approximately 100kDa in the four vectors containing the 45Å-spacer. There, the spacer might increase accessibility of the NANP-repeat, thus enhancing the propensity for dimerization inherent to the CS protein [34], which is also supported by the observation that under non-reducing conditions, the 50 kDa band decreases and the 100 kDa band increases in intensity (data not shown). This, however, remains speculative since no obvious differences in decoration intensity between the vectors containing the 45Å-spacer or the direct pIX-CS_short_ fusion were observed in the EM analysis.

The detection of pIX-display variant expression under non-replicating conditions in A549 cells is consistent with previous observations [31] and might be due to the activity of the pIX promoter itself or possible enhancing effects of the CMV promoter directly upstream of the pIX region. However, the consequences of possible pIX-antigen fusion *in vivo* expression, in addition to capsid-display, on the induction of antibody responses remain to be determined.

In agreement with previous observations in HAdV5 vectors, our pIX-modified HAdV35 vector preparations exhibited the same heat instability as vectors lacking pIX (ΔpIX) [23]. Despite this instability, all pIX-display modified HAdV35 vectors were comparable to the pIX-unmodified vectors in terms of physical viral particle yields, infectious virus titer, vector infectivity and productivity. Overall we conclude that the fusion of the CS_short_ model antigen by itself or in combination with the linker and/or spacer to pIX did not interfere with pIX capsid-incorporation, the transgene-expression in A549 cells or productivity in the producer cell line.

Immunological characterization of the vectors demonstrated that surface-display of CS_short_ on HAdV35 vector particles significantly increased the potency of the model antigen to induce CS-specific antibody titers over those induced by the CS-transgene expressed from HAdV35.CS, or by soluble CS protein. Compared to immunization with soluble protein, fusion of the CS_short_ antigen to pIX may ensure presentation of the antigen in its native conformation and in a highly repetitive nature inherent to viral capsids, both of which were shown to deliver strong activation signals to the recognizing B-cells [35, 36]. Accessibility of the antigen on the surface of the AdV vector particle may be of additional importance, as demonstrated by the increased humoral responses induced by the pIX-CS_short_ fusion constructs including the glycine-linker and/or 45Å-spacer in comparison to the direct fusion construct. The glycine-linker may be acting as a molecular hinge, making the rigid, rod-shaped NANP-repeat domain of the CS protein more flexible in its interaction with the two antigen-binding domains of the B-cell receptor. By lifting the antigen up towards the surface of the hexon molecules, the 45Å-spacer molecule may ensure display of the antigen over its full length at an optimal distance from the surface of the AdV capsid. However, this effect may be dependent on length and conformation of the linker and/or the antigen [25].

We further showed that mixing soluble CS protein with unmodified, empty AdV vector particles failed to match responses reached by pIX-modified vectors or adjuvanted CS protein. AdV particles have previously been reported to mediate a strong adjuvant-effect on co-administered lipopeptide antigens that is independent of their vector function and likely mediated by capsid components [32]. AdV vectors induce the release of pro-inflammatory cytokines [37, 38], including type-I interferons, which enhances immune responses to peptides [39] or vector-encoded transgenes [40]. Although HAdV26- and HAdV35-based vectors induce higher levels of innate cytokine responses than HAdV5-based vectors [41], in our study all HAdV vectors (HAdV26, HAdV35, HAdV5) mixed with CS protein equally failed to increase CS-specific antibody responses, indicating that potential adjuvant-effects of AdV vector particles on protein-based vaccines are likely independent of innate cytokine responses but depend on the biophysical properties of the mixed protein; for example, its propensity to adhere to the surface of the viral particle. Therefore, our findings indicate that the high immunogenicity of pIX-modified display-vectors is related to a physical association of the CS_short_ antigen with the viral particles.

Our findings of increased humoral immune responses in addition to strong cellular responses induced by AdV vectors corroborate those reported by Bayer et al. [25], who demonstrated immunogenicity and efficacy of simultaneous pIX-display and expression of the gp70 envelope protein of Friend Murine Leukemia virus. AdV vectors are known to induce high levels of antigen-specific IFNγ-producing CD8+ T-cells [42] and combining them in a prime-boost regimen with other vaccine types has proven efficient in eliciting strong T-cell as well as humoral responses [43–46]. We previously demonstrated that HAdV35-based vectors encoding the CS antigen are highly effective in improving the T-cell responses induced by a CS protein prime in a heterologous prime-boost schedule [10]. Here we demonstrate that implementing pIX-display technology on CS-transgene expressing HAdV35 vectors induces strong antibody and IFNγ-producing CD8+ T-cell responses after a single immunization. It remains to be determined whether homologous prime-boost with the pIX-modified display-/expression vectors would further increase the magnitude of the immune responses, and whether this could serve as a potent, simplified regimen compared to heterologous prime-boost regimens.

In conclusion, we demonstrated that pIX-display technology is highly immunogenic and offers the opportunity to induce both T- and B-cell responses using a simplified vaccination regimen. Moreover, the pIX-CS_short_ display-vectors can be efficiently produced at high viral particle yields that are in the same range as the conventional transgene-expression vectors. Considering that many of the pathogens which require a complex immune response for protection are endemic to resource-limited settings, simplification of vaccine regimen and high yield makes pIX-display technology particularly valuable to develop vaccines for underserved communities where they may have the highest impact.

## Supporting information

S1 Fig pIX-CS_short_ capsid incorporation in purified HAdV35 vector preparations using the anti-pIX antibody. To confirm capsid incorporation of the pIX-CS_short_ variants (~50 kDa), purified HAdV35 vector preparations (1.5 x10^10^ VP/well) were analyzed by Western blot using the monoclonal anti-pIX antibody. To ensure equal loading the blots were also stained with anti-fiber antibody (~35 kDa). Marker (M) is indicated with the corresponding kDa band size. An additional band (~100 kDa) is indicated with an asterisk (*). The pIX-fusion proteins migrate higher than their predicted size in kDa due to the NANP-repeat in the CS protein, a feature probably also affecting the detection of the pIX-CS_short_ and pIX-Gly-CS_short_ variants due to anti-pIX epitope masking (single epitope).
(TIF)

S2 Fig CS-specific antibodies induced by pIX-CS_short_ displayed on HAdV35 vectors recognize native CS on *P.falciparum* slides. Binding of CS-specific IgG in pooled sera of Balb/C mice 6 weeks after immunization with 1x10^10^ VP of the indicated vectors. Images taken at 40-fold magnification. Left panels show the white light (white arrows indicate sporozoite location), middle panels the fluorescence and the right panels show the merged images. White bars correspond to 20μ`m length.
(TIF)

S1 Table HAdV35 pIX-display vectors: overview of pIX capsid incorporation and CS transgene expression in A549 overview cells (DOCX)

S2 Table Overview of HAdV35 pIX-CS_short_ display-vectors: viral titers and producibility as determined by optical density (DOCX)

S3 Table Statistical significance in differences in CS-specific serum IgG titers elicited by the three pIX-CS_short_ modified HAdV35.empty vectors compared to HAdV35.CS (corresponding to Fig 3A). (DOCX)
