# Supplementary figures and images for: Antigen capsid-display on human adenovirus 35 via pIX fusion is a potent vaccine platform
Source: PLoS One. 2017 Mar 31;12(3):e0174728. doi: 10.1371/journal.pone.0174728 (PMC5375148; doi:10.1371/journal.pone.0174728)

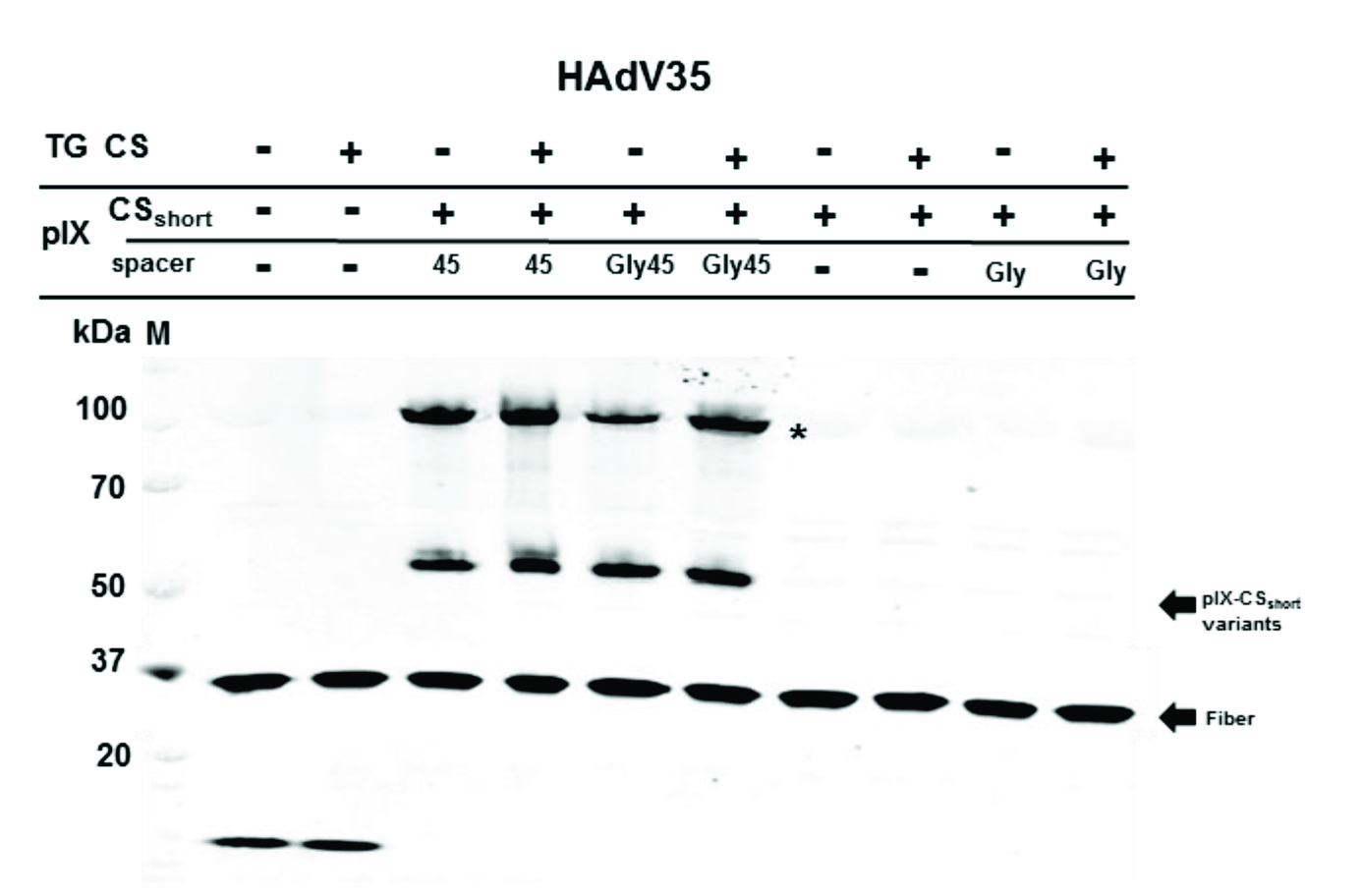

Supplement: S1 Fig — To confirm capsid incorporation of the pIX-CSshort variants (~50 kDa), purified HAdV35 vector preparations (1.5 x10^10^ VP/well) were analyzed by Western blot using the monoclonal anti-pIX antibody. To ensure equal loading the blots were also stained with anti-fiber antibody (~35 kDa). Marker (M) is indicated with the corresponding kDa band size. An additional band (~100 kDa) is indicated with an asterisk (*). The pIX-fusion proteins migrate higher than their predicted size in kDa due to the NANP-repeat in the CS protein, a feature probably also affecting the detection of the pIX-CSshort and pIX-Gly-CSshort variants due to anti-pIX epitope masking (single epitope). (TIF) [file pone.0174728.s001.tif]

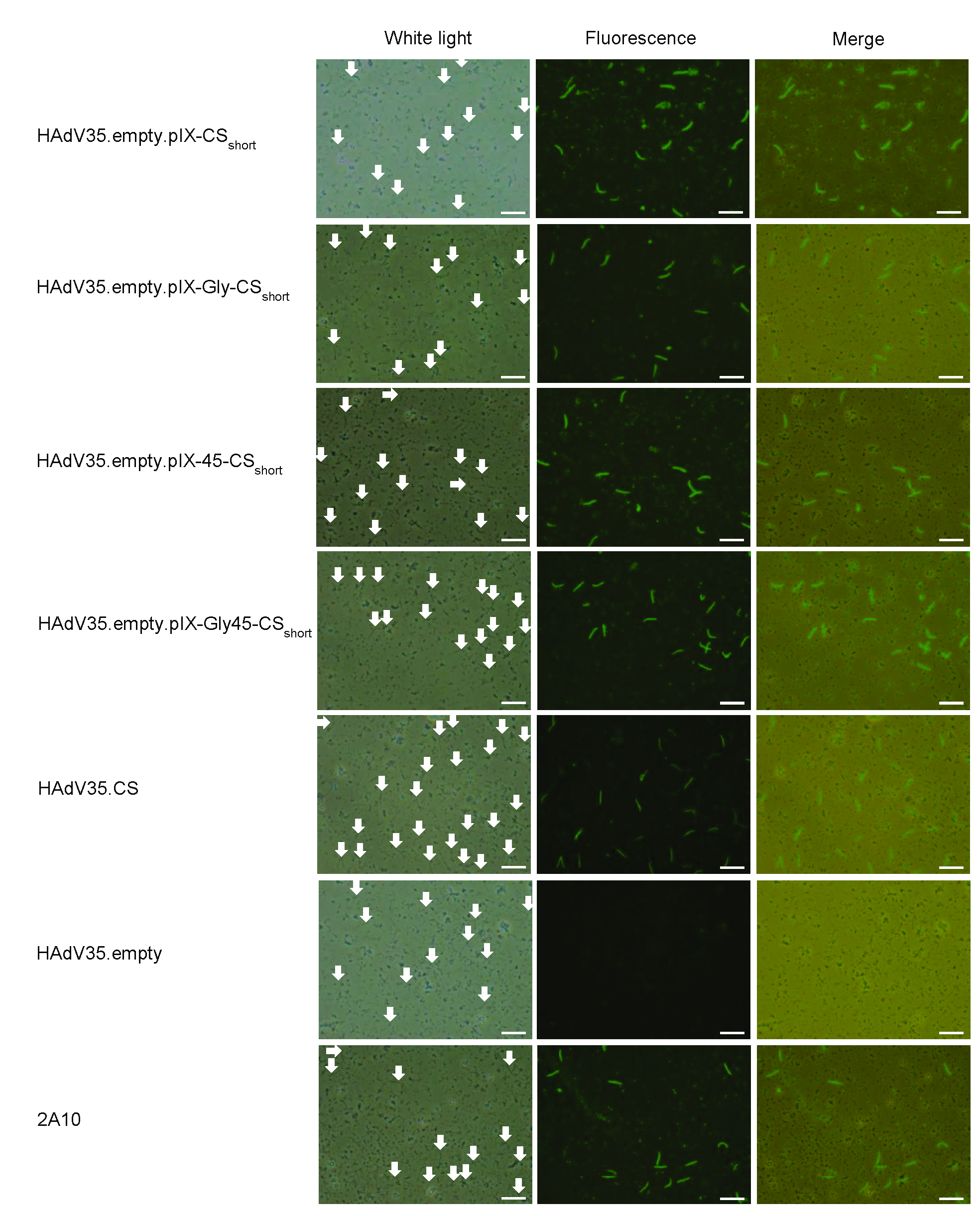

Supplement: S2 Fig — Binding of CS-specific IgG in pooled sera of Balb/C mice 6 weeks after immunization with 1x10^10^ VP of the indicated vectors. Images taken at 40-fold magnification. Left panels show the white light (white arrows indicate sporozoite location), middle panels the fluorescence and the right panels show the merged images. White bars correspond to 20μ`m length. (TIF) [file pone.0174728.s002.tif]
