# Supplementary material for: Antigen capsid-display on human adenovirus 35 via pIX fusion is a potent vaccine platform
Source: PLoS One. 2017 Mar 31;12(3):e0174728. doi: 10.1371/journal.pone.0174728 (PMC5375148; doi:10.1371/journal.pone.0174728)
Supplement: S1 Table — (DOCX) [file pone.0174728.s003.docx]

**S1 Table: HAdV35 pIX-display vectors: overview of pIX capsid incorporation and CS transgene expression in A549 cells**

|  |  | |  | **pIX-CS_short_**  **capsid incorporation** | | | | **pIX-CS_short_**  **expression in producer cell line** | | **CS**  **transgene expression** |
| --- | --- | --- | --- | --- | --- | --- | --- | --- | --- | --- |
|  |  | |  | **WB** | | | **EM** |  |  |  |
| **Vector** | **TG** | | **pIX-modification** | **ɑ-pIX** | | **ɑ-CS** | **ɑ-CS** | ɑ-pIX | ɑ-CS | **ɑ-CS** |
| **HAdV35** | CS | | n/a | **+** | | n/a | **-** | - | + | **+** |
|  | Empty | | n/a | **+** | | n/a | **-** | + | - | n/a |
|  | CS_short_ | | n/a | n/d | | n/d | n/d | + | - | **-** |
|  | Empty | | 45-CS_short_ | **+** | | **+** | **+** | + | - | n/a |
| _#_ | CS | | 45-CS_short_ | **+** | | **+** | **+** | + | + | **+** |
|  | Empty | | Gly45-CS_short_ | **+** | | **+** | **+** | + | - | n/a |
|  | CS | | Gly45-CS_short_ | **+** | | **+** | n/d | + | + | **+** |
|  | Empty | | GlyCS_short_ | **-** | | **+** | **+** | - | - | n/a |
|  | CS | | GlyCS_short_ | **-** | | **+** | n/d | - | + | **+** |
|  | Luc | | GlyCS_short_ | n/d | | **+** | n/d | - | - | n/a |
|  | Empty | | CS_short_ | **-** | | **+** | **+** | - | - | n/a |
|  | CS | | CS_short_ | **-** | | **+** | **+** | - | + | **+** |
|  | Empty | | CS | **-** | | **-** | n/d | **+** | **-** | n/a |
|  | CS | | CS | **-** | | **-** | n/d | **+** | **+** | **+** |
|  | |  | | | n/a: not applicable, (-) not detected, n/d: not determined, # no batch-to-batch differences observed, WB: Western blot, EM: electron microscopy, CS: circumsporozoite protein, TG: transgene in E1 | | | | | |
