# Supplementary material for: Antigen capsid-display on human adenovirus 35 via pIX fusion is a potent vaccine platform
Source: PLoS One. 2017 Mar 31;12(3):e0174728. doi: 10.1371/journal.pone.0174728 (PMC5375148; doi:10.1371/journal.pone.0174728)
Supplement: S2 Table — (DOCX) [file pone.0174728.s004.docx]

**S2 Table: Overview of HAdV35 pIX-CS_short_ display vectors: viral titers and producibility as determined by optical density**

| **Vector** | **TG** | **pIX modification** | **VP/ml** | **IU/ml** | **VP/IU ratio** | **VP/cm_2_** |
| --- | --- | --- | --- | --- | --- | --- |
| **HAdV35** | CS |  | 1.9x10^12^ | 5x10^11^ | 3 | n/d |
|  | Empty |  | 1.0x10^12^ | 3.3x10^11^ | 3 | 6.1x10^8^ |
|  | Empty | 45-CS_short_ | 1.4x10^12^ | 1.8x10^11^ | 8 | 2.5x10^8^ |
| _#_ | CS | 45-CS_short_ | 2.8x10^11^ | 1.7x10^10^ | 17 | 1.3x10^8^ |
|  | Empty | Gly45-CS_short_ | 1.1x10^12^ | 6.0x10^10^ | 19 | 6.3x10^8^ |
|  | CS | Gly45-CS_short_ | 1.9x10^12^ | 1.8x10^11^ | 11 | 1.8x10^9^ |
|  | Empty | GlyCS_short_ | 3.3x10^12^ | 2.9x10^11^ | 11 | n/d |
|  | CS | GlyCS_short_ | 2.8x10^12^ | 2.0x10^11^ | 13 | n/d |
|  | Luc | GlyCS_short_ | 4.8x10^11^ | 4.9x10^10^ | 10 | n/d |
|  | Empty | CS_short_ | 2.6x10^12^ | 4.2x10^11^ | 6 | n/d |
|  | CS | CS_short_ | 1.5x10^12^ | 2.0x10^11^ | 8 | 8.8x10^8^ |
| CS: circumsporozoite protein  TG: transgene in E1  n/d: not determined, # second batch comparable titers  VP: physical viral particles  IU: infectious units  VP/IU: viral particle to infectious units ratio  VP/cm^2^: viral particles generated per cm^2^ production- flask | | | | | | |
