# Supplementary material for: Antigen capsid-display on human adenovirus 35 via pIX fusion is a potent vaccine platform
Source: PLoS One. 2017 Mar 31;12(3):e0174728. doi: 10.1371/journal.pone.0174728 (PMC5375148; doi:10.1371/journal.pone.0174728)
Supplement: S3 Table — (DOCX) [file pone.0174728.s005.docx]

**S3 Table: Statistical significance in differences in CS-specific serum IgG titers elicited by the three pIX-CS_short_ modified HAdV35.empty vectors compared to HAdV35.CS (corresponding to figure 3A).**

|  |  | **p-value*** |  |  |
| --- | --- | --- | --- | --- |
|  | **10^7^ VP/mouse** | **10^8^ VP/mouse** | **10^9^ VP/mouse** | **10^10^ VP/mouse** |
| HAdV35.epmty.pIX-CS_short_ | n/d | 0.1169 | 0.6661 | 0.0658 |
| HAdV35.epmty.pIX-45-CS_short_ | n/d | **0.000** | **0.0366** | **0.0029** |
| HAdV35.epmty.pIX-Gly-CS_short_ | n/d | **0.003** | **0.0082** | 0.6378 |
| HAdV35.epmty.pIX-Gly45-CS_short_ | n/d | **0.0002** | 0.227 | **0.0007** |

n/d: not done (indicated dose excluded from statistical analysis since no titers above lower limit of quantification for the CS-IgG ELISA assay were elicited).

* Adjusted p-values for the titers elicited over time by the indicated vectors compared to HAdV35.CS given at the same dose (fixed reference group). P-values ≤ 0.05 were considered significant and are indicated in bold.
